# Epigenetic Modifications in Alzheimer’s Neuropathology and Therapeutics

**DOI:** 10.3389/fnins.2019.00476

**Published:** 2019-05-10

**Authors:** Michelle Esposito, Goldie Libby Sherr

**Affiliations:** ^1^Department of Biology, Georgian Court University, Lakewood, NJ, United States; ^2^Department of Biology, College of Staten Island, City University of New York, New York, NY, United States; ^3^Department of Biological Sciences, Bronx Community College, City University of New York, New York, NY, United States

**Keywords:** epigenetics, Alzheimer’s, histone acetylation, methylation, chromatin remodelers, HDACs

## Abstract

Transcriptional activation is a highly synchronized process in eukaryotes that requires a series of *cis*- and *trans*-acting elements at promoter regions. Epigenetic modifications, such as chromatin remodeling, histone acetylation/deacetylation, and methylation, have frequently been studied with regard to transcriptional regulation/dysregulation. Recently however, it has been determined that implications in epigenetic modification seem to expand into various neurodegenerative disease mechanisms. Impaired learning and memory deterioration are cognitive dysfunctions often associated with a plethora of neurodegenerative diseases, including Alzheimer’s disease. Through better understanding of the epigenetic mechanisms underlying these dysfunctions, new epigenomic therapeutic targets, such as histone deacetylases, are being explored. Here we review the intricate packaging of DNA in eukaryotic cells, and the various modifications in epigenetic mechanisms that are now linked to the neuropathology and the progression of Alzheimer’s disease (AD), as well as potential therapeutic interventions.

## Introduction

Alzheimer’s disease (AD) is a neurodegenerative disease characterized by significant impairments in neural synapses and deficiencies in memory. Generally, it is a disease that starts off gradually, with the most common hallmark symptom being that of short-term memory loss (Burns and Iliffe, 2009). AD deteriorates progressively, with symptoms intensifying over the passage of time. These symptoms often include extensive memory loss, confusion, difficulty with language, mood swings, and behavioral issues (Burns and Iliffe, 2009). In most cases, AD progresses to dementia, and ultimately leads to death, frequently from bronchopneumonia or acute cerebrovascular incidents (Mölsä et al., 1986).

The classic AD symptoms come alongside debilitating neural atrophy. A depletion in both the number of neurons and synapses in the brain are characteristic trademarks of AD (Hamos et al., 1989; Spangenberg and Green, 2017). The deficiency of these key brain cells and nerve connections often leads to the deterioration of both the temporal and parietal lobes of the brain, as well as parts of the frontal cortex and cingulate gyrus, as well as brainstem nuclei (Wenk, 2003; Huang et al., 2007; Braak and Del Tredici, 2012). Research using MRI imaging has shown that patients with AD have an actual physical decrease in the size of specific brain regions as a result of this neuronal loss (Callen et al., 2001).

In addition to neuron and synapse deterioration, patients with AD have a greater buildup of amyloid plaques and neurofibrillary tangles in the brain compared to those without AD (Tiraboschi et al., 2004). The atypical accumulation of these substances is usually found in the specific areas of the brain associated with AD, such as the temporal lobe (Bouras et al., 1994). Amyloid plaques consist of amyloid beta peptides, which are fragments of the amyloid precursor protein (APP). APP, a transmembrane protein of the neuronal membrane, is crucial for neuron growth, repair, and overall function (Turner et al., 2003; Priller et al., 2006) In AD, the γ-secretase and β-secretase proteolytic cleavage, unlike α-secretase cleavage, can yield amyloidogenic processing that can lead to substantial neurpathologies (Kang et al., 1987; Hooper, 2005; Zhang et al., 2011). For instance, γ-cleavage at C83 or C89 can yield amyloid β (Aβ) peptides, Aβ40 and Aβ42, which are main constituents of neuropathological plaques in AD brains (Zhang et al., 2010). These Aβ peptides then form packed deposits that accrue outside the neuron and surround it Tiraboschi et al. (2004) and Zhang et al. (2011). While the buildup of these plaque masses is a clear hallmark of AD, the exact mechanism of how this accumulation of beta amyloid peptides lead to the pathology of Alzheimer’s is still unclear (Van Broeck et al., 2007).

Additionally, Tau, a microtubule associated protein, also accumulates abnormally in the brains of people with AD. While the tau protein normally functions to stabilize the microtubes of a cell’s cytoskeleton in its phosphorylated state, tau becomes hyperphosphorylated in AD. This hyperphosphorylated tau combines with other threads to form neurofibrillary tangles. These tangles accrue inside the neuron and greatly impact normal transport (Hernandez and Avila, 2007). The elevated presence of both these tangles as well as the above-mentioned amyloid plaques in the brain have been key indicators of AD.

While the trademark pathology and symptoms of AD are well-known, the underlying pathways which lead to the disease are not. Currently, there is no known cure to treat AD (Holtzman et al., 2011; Lindsley, 2012; Mitra et al., 2019). However, recent findings indicate that epigenetic modifications are fundamental in the process of regulating gene expression, particularly for that of memory (Kosik et al., 2012). Furthermore, AD has been shown to exhibit an epigenetic blockade, or a widespread decline in gene expression, that is thought to be influenced by post-translational histone modifications (Sananbenesi and Fischer, 2009; Gräff et al., 2012). Taken together, it seems that epigenetics may play a greater role in AD than previously thought. Thus, by better understanding and studying the impairments of epigenetic modifications in AD, potential new therapies to treat the disease can be designed.

Epigenetics is defined as the study of phenotypic changes that occur from the modification of chromatin without changes to the actual DNA sequence (Dupont et al., 2009). In order to understand how epigenetics work, it is important to understand how DNA is packaged. The genome of a eukaryotic organism consists of a vast amount of genetic information that must be stored in the nucleus of each cell (Kornberg, 1974; Peterson and Laniel, 2004). Since these lengthy DNA molecules are quite extensive in size, they must be intricately packaged into higher ordered structures so that they can fit into the relatively small sized nucleus. In order to accomplish this feat, the DNA is wound around histone proteins, which associate with each other via electrostatic and hydrogen interactions, and thus create the structural unit termed the nucleosome (Cairns, 2009). A nucleosome consists of the eight core histone proteins, H2A, H2B, H3, and H4, each of which is present as a pair, and the DNA that wraps around them (Kornberg and Lorch, 1999; Pérez-Martìn, 1999; Rando and Winston, 2012). A histone tail consisting of amino acid chains that are abundant in positively charged residues, extend from the histone core (Pérez-Martìn, 1999; Martin and Zhang, 2005). There is also an H1 histone protein, called the linker histone, which associates with the linker DNA. The linker DNA are the regions of DNA that exist between nucleosomes after they are structured into the higher ordered string-like chromatin (Happel and Doenecke, 2009; Harshman et al., 2013). These regions are extremely crucial for gene expression and regulation (Clapier and Cairns, 2009).

**FIGURE 1:**
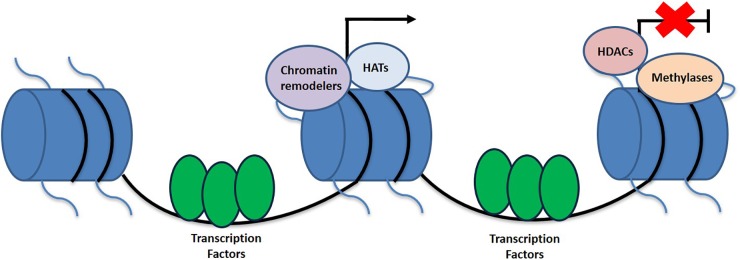
Schematic representation of histone linkage and epigenetic variables that contribute toward the activation and deactivation of genes. Epigenetic modifications contributing to transcriptional activation include HATs and chromatin remodelers, while HDACs and methylation are more associated with the silences of gene expression.

While the higher ordered chromatin structure allows for the DNA to be tightly packaged and fit into the nucleus, it produces its own set of issues. Due to the condensed form of DNA, the promoter region is not as easily accessible for important cellular processes that require the DNA template (Smith and Peterson, 2005; Morrison and Shen, 2009). In order to allow for the promoter to be reachable, nucleosome structure must be altered or disrupted. The two main categories of enzymes that specifically target nucleosomes for this purpose include those that covalently modify histone proteins, such as those that carry out acetylation, deacetylation, and methylation and those that hydrolyze ATP to reposition the nucleosome and thus conduct chromatin remodeling (Margueron et al., 2005; Smith and Peterson, 2005) (Figure 1). Additionally, there are other types of histone modifications as well including that of phosphorylation, ubiquitination, sumoylation, and ADP ribosylation amongst others.

### Acetylation and Deacetylation

The first two types of epigenetic modifications in this review are that of histone acetylation and deacetylation. Histone acetylases, or HATs, belong to the category of enzymes that covalently modify histone proteins by carrying out acetylation on lysine residues of the core histone tails (Marmorstein, 2001; Roth et al., 2001). Acetylation is a histone modification often associated with transcriptional activation. There are two main type of HATs: Type A and Type B. Type A HATs are localized in the nucleus and act on the histones associated with the chromatin (Kornberg and Lorch, 1999; Roth et al., 2001). Type B HATs however are in the cytoplasm and have been found to act on freshly synthesized histones that have not yet been associated with chromatin (Brownell and Allis, 1996; Roth et al., 2001; Parthun, 2007). Histone deacetylases, or HDACs, are yet another group of enzymes that covalently modify histone proteins. While HATs are responsible for neutralizing histone tails by acetylating lysine residues, HDACs counter their effects by deacetylating lysine residues. They are therefore associated with condensing chromatin and gene repression since they directly revert the histone tails back to their charged status (Pazin and Kadonaga, 1997). In mammals, there are four classes of histone deacetylases: classes I, II, III, and IV, and their classification is based on multiple factors including that of function and DNA sequence. Depending on the type of HDAC, these deacetylases can be found both in the nucleus and cytoplasm of the cell (De Ruijter et al., 2003).

### DNA Methylation and Histone Methylation

While histone acetylation, histone deacetylation, histone methylation, and chromatin remodeling are all key players in influencing the epigenetics of an organism, there is yet another crucial mechanism that is involved in the epigenetic code. DNA methylation is a process that adds methyl groups to the DNA structure. Methylation can occur on both cytosine and adenine bases. While cytosine methylation is quite common in mammals, it should be noted that methyl groups on adenine bases have recently been detected in mammalian cells as well (Wu et al., 2016). Cytosine methylation involves the addition of methyl groups onto cytosine bases that come directly before guanine bases on the DNA strand. These are called CpG dinucleotides (Bird, 1986). Interestingly, recent research has proposed the importance of DNA methylation in long term memory, a crucial indication of its potential relationship to AD (Miller and Sweatt, 2007; Day and Sweatt, 2010).

In addition to DNA methylation, histone methylation is also significant. Histone methyltransferases, or HMTs, are enzymes that methylate lysine or arginine residues on the histone tails of histones H3 and H4. HMTs have been linked to both gene activation and repression. There are two main families of HMTs, and they are categorized based on the residues they methylate. The first group consists of histone lysine methyltransferases, which are the HMTs that methylase lysine. The second group is made up of protein arginine methyltransferases, which are responsible for methylating arginine (Wood and Shilatifard, 2004).

### Chromatin Remodelers

While the above three epigenetic mechanisms refer to groups of enzymes that covalently modify histones, chromatin remodeling complexes are enzymes that belong to a category all of their own. These enzyme complexes utilize ATP to reposition the nucleosome and literally change the dynamics of the chromatin structure by modifying the connections between the DNA and histone proteins (Tsukiyama et al., 1999). This process is achieved through various mechanisms including that of nucleosome sliding, nucleosome repositioning, and ejection (Fazzio and Tsukiyama, 2003; Mohrmann and Verrijzer, 2005; Cairns, 2007, 2009; Clapier and Cairns, 2009). There are a number of families of chromatin remodelers in eukaryotic cells. These include the families of SWI/SNF, ISW1, NuRD/Mi-2 CHD, INO80, and SWR1. All of these groups of chromatin remodelers are similar in their ATPase domain, though they do differ in their specific remodeling functions (Lusser and Kadonaga, 2003; Kim et al., 2006).

Due to the significance of epigenetics, and its suggested involvement in the regulation of gene expression, particularly for that of memory, it is no wonder that connections to its role in AD have been made. In this review, we will discuss the epigenetic dysregulation observed in AD with an emphasis on the potential of epigenetic therapies to target the neuropathology exhibited as the disease progresses.

## HAT/HDAC Implications in AD Therapeutics

Learning and memory involve intricate coordination amongst a network of various factors and pathways. Long-term memory and synaptic plasticity are dependent upon activations beyond the early induction phases of gene expression, which suggests a wide-ranging potential for epigenetic interplay (Pittenger and Kandel, 2003). Histone acetylation (and its counterpart, deacetylation; HATs and HDACs) is one of many epigenetic mechanisms now identified as playing a significant role in long-term potential (LTP) and memory formation, observable through fear conditioning and spatial memory exercises (Rogan et al., 1997; Francis et al., 2009).

**FIGURE 2:**
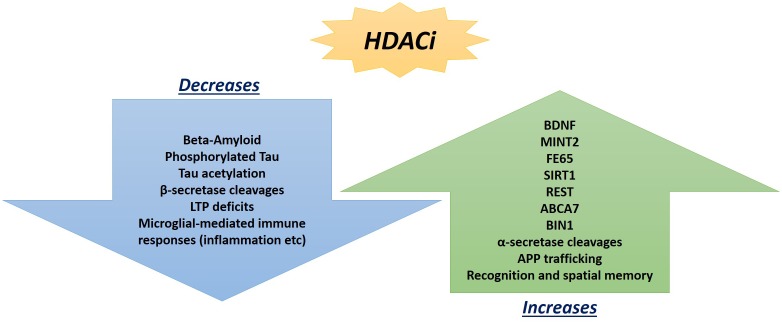
Promising beneficial consequences of HDAC inhibitor treatments observed so far in AD mouse models and post-mortem hippocampal analyses that can ameliorate neuropathologies.

Hippocampal LTP is an *N*-methyl-D-aspartate glutamate receptor-dependent response involving a continuing increase in synaptic potentiation maintained for longer than 1 h, which serves as the leading model of synaptic plasticity and learning in mammalian models (Bliss and Lomo, 1973; Bliss and Collingridge, 1993). Previous focus on epigenetic factors in LTP only analyzed methylation, and neglected the significant impact HATs and HDACs can play, particularly the potential of HDAC inhibitors (Kazantsev and Thompson, 2008). Although once regarded for their potential in cancer treatments (Vigushin and Coombes, 2002), HDAC inhibitors are now regarded as potential therapeutic targets in AD patients (Xu et al., 2011) with a wide array of effects (Figure 2).

Alzheimer disease is linked to variants in amyloid-β-protein precursor (*APP*), presenilin-1, and presenilin-2 (*PS1* and *PS2*) genes (Yan et al., 1995; Barglow and Cravatt, 2007), which lead to neuropathological characteristics of advanced accumulation of β-amyloid in the brain and the dyruption of synaptic LTP transmissions (Shankar et al., 2008). Fear conditioning training using *APP/PS1* mice has demonstrated decreased contextual freezing performance could be restored back to wild type levels via acute treatment with Trichostatin A (TSA), an HDAC inhibitor. *APP/PS1* chimeric mutant mouse/human transgenes result in AD phenotypes with β-amyloid plaque deposits accumulating by 6 months of age (Jankowsky et al., 2003). In the deficient phenotype mice, hippocampal acetylated H4 levels were approximately half that of WT littermates. HDAC inhibitor treatment then allowed for the restoration of normal higher H4 acetyl levels comparable to WT litters. Overall, TSA treatment rescued H4 acetylation levels, contextual freezing times, and deficits in hippocampal LTP, as observed through tetanic stimulations and contextual spatial learning) (Francis et al., 2009). Further studies have since expanded the HDAC drugs utilized with similarly significant results. HDACi drugs, sodium valproic acid, as well as Suberoylanilide hydroxamic acid (SAHA) and Sodium Butyrate (NaB), have been shown to yield significantly higher freezing levels in standard electric footshock freezing fear conditioning compared to their vehicle-treated (control) mutant *APP/PS1* littermates. Treatment restored AD phenotypes to results that not only were no longer significantly different from WT littermates, but also were maintained even weeks later and did not modify any other aspects of behavior not related to AD pathology, such as exploratory nature or immediate freezing responses (Kilgore et al., 2010). That longevity of effect is critical in any therapeutic marketable compound, and has since been explored to maximize the significant impact that these drugs can have for patients. Two HDAC inhibitors with longer half-life and greater Blood Brain Barrier penetration have been developed. A mercaptoacetamide-based class II HDACi and a hydroxamide-based class I and II HDACi both decrease β-amyloids *in vitro* by reducing gene expression of components and increasing degradation enzyme gene expression, which ultimately rescued learning and memory defects in AD mice while decreasing tau (Sung et al., 2013).

Beyond standard learning deficits, AD can also manifest in seizures and epileptic episodes, which further instigate cognitive decline. These seizures increase *ΔFosB* transcription factor expression, which in turn recruits HDAC1 in the hippocampus to suppress *c-Fos*, a protooncogene known for its role in memory and synaptic plasticity (Saura et al., 2004). HDAC inhibition of *ΔFos* in *APP* mutant AD mice via 4-phenylbutyric acid (Class I HDAC 4-PBA) or MS-275 (inhibitor of HDAC1-3) has now been shown to reverse the suppression of *c-Fos* and thus increases cognition performance in AD mice as observed with object location memory tasks and hippocampus-dependent spatial memory tasks (Corbett et al., 2017).

Another transcription factor known to have significance in AD pathology that may benefit from epigenetic therapeutic interventions is *PU.1*, which is crucial in the development of myeloid cells and microglia gene expression (Rustenhoven et al., 2018). Genome-wide association studies shows that reductions in *PU.1* is a factor in delaying the onset of AD (Huang et al., 2017). Microarray analyses, RT-qPCR and immunocytochemistry of *PU.1* knock-downs have demonstrated modified AD-associated microglial genes that are known to be involved in both, innate and adaptive immunity. Further high-throughput drug screenings with FDA-approved drugs have yielded the identification of HDAC-inhibitor, Vorinostat, as efficient in attenuating *PU.1* expression in human microglia. Combined results of these analyses suggested Vorinostat or other HDAC inhibitors that knockdown *PU.1* expression may be useful as potential therapies that could reduce microglial-mediated immune responses, such as the excess inflammation observed in AD (Rustenhoven et al., 2018; Smyth et al., 2018).

**FIGURE 3:**
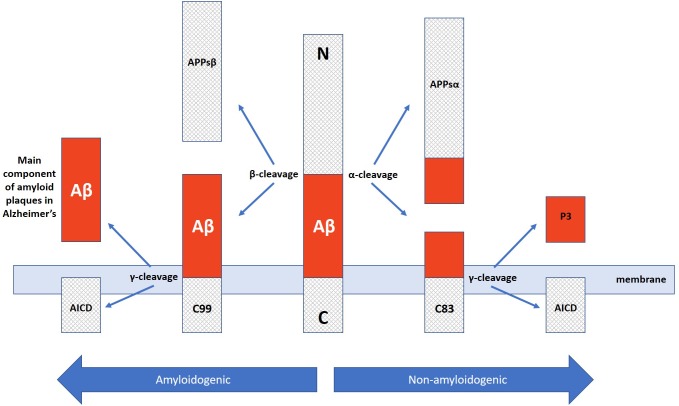
Amyloidogenic and non-amyloidogenic pathway of APP cleavage via α and β secretases in which the amyloidogenic path yields neuropathological Aβ plaque peptides critically involved in Alzheimer’s disease.

Along those lines, it is important to once again emphasize that AD presents with a wide range of pathologies and thus, one single target may not suffice to ameliorate the deficits exhibited across the board. Instead, it may be of greater promise to explore multitargeting therapeutics. One study has already exhibited promising results with this technique by utilizing a single drug, HDACi M344, to affect the expression of multiple AD-related genes. M344 has been shown to decrease β-amyloid, phosphorylated tau, β-secretase, and *APOE𝜀4*, while it also increased *ADAM10*, as well as increased *BDNF*, *MINT2*, *FE65*, *SIRT1*, *REST*, *ABCA7*, *BIN1*, and APP trafficking (Volmar et al., 2017). This is significant as β-secretase (as well as γ-secretase) cleaves Amyloid Precursor Protein (APP) (Figure 3) in a way that leads to neuropathologies in AD such as senile plaques, neuroplasticity deficits, and tau hyperphosphorylation (Nistor et al., 2007). If instead cleavage is performed by α-secretase (ADAM10), then amyloidogenic processing is avoided and neuropathology does not present (Colciaghi et al., 2002). Ultimately, mice treated with M344 exhibited significant cognitive benefits in recognition and spatial memory testing (Volmar et al., 2017), which demonstrates the potential to utilize a multitargeting drug to resolve the polygenic aspect of AD and other neurodegenerative faults. Another example of how multitargeting can be of value in AD therapy is the development of a novel “first-in-class” small molecule called CM-414 that ties HDAC inhibition with PDE5 inhibition, both of which individually have shown auspicious results (Cuadrado-Tejedor et al., 2017). PDE 5 inhibition (as seen with vasodilator, Viagra) improves AD phenotype deficits, as it is a molecule that increases phosphorylation of CREB, which is a key player in memory. Long-lasting improvement of synaptic function, CREB phosphorylation, as well as the reversal of memory deficits, cGMP/PKG/pCREB signaling deficits, and neuroinflammation, while creating a long-lasting decrease in Amyloid-beta levels have all been observed with PDE5 inhibition (Puzzo et al., 2009; Zhang et al., 2013). PDE5 is ultimately involved in degradation of cGMP in various locations including brain tissues. The nitric oxide/cGMP/CREB pathway is critical to learning/memory process, so degradation of cGMP is implicated in neurodegenerative nature of AD and drugs inhibiting this degradative process are thus promising therapeutics (Fiorito et al., 2013). Combining this effectiveness with the effectiveness of HDAC inhibition can amplify the benefits for patients. Chronic treatment with the dual inhibitor CM-414 is capable of rescuing deficient LTPs in *APP/PS1* mice, while also reducing the beta-amyloid and phosphorylated tau levels. Furthermore, CM-414 has been shown to increase the inactive form of Glycogen synthase kinase-3β (GSK3β) (Cuadrado-Tejedor et al., 2017). GSK3β is a kinase involved in microtubule stability and cognition with its connection to the phosphorylation of tau (Bhat and Budd, 2002) and thus is associated with the neuropathology of AD (Pláteník et al., 2014). Additionally, CM-414 has resulted in a decrease in dendritic spine density on hippocampal neurons, as well as reversed cognitive deficits observed through fear conditioning testing and Morris water maze test spatial memory testing as it induces synaptic gene expression. The *in vitro* and *ex vivo* activity of the drug has been quite promising as it demonstrates how beneficial it can be to use multiple-target therapies based on the complex and multifactorial nature of AD neuropathology (Cuadrado-Tejedor et al., 2017). The only concern with this, however, is that increased targets means an increased risk of additional side effects, as has been observed when Vorinostat leads to severe diarrhea and anorexia when it has been utilized in higher doses during carcinoma treatment studies (Ree et al., 2010).

Testing expanding beyond mouse models has also been quite promising with regard to HDAC inhibition drugs. Repeated treatment of triple transgenic AD mice with RGFP-966 has been shown to decrease β-amyloid protein levels, reversed the phosphorylation of tau, and led to improved spatial learning and memory results as tested by open-field, balance beam, treadmill, and nest-building behavioral analyses. RGFP-966 was further shown to increase BDNF expression and decrease tau phosphorylation and tau acetylation, while also reducing the neuropathology-inducing β-secretase cleavage of APP. RGFP-966 testing was then expanded to explore the impact when applied to induce pluripotent stem-cell-derived primary neurons from AD patients. Although it was a minimal sample size of only two patients compared to two healthy controls, the results were promising with a rescue of AD pathology with a decrease in beta-amyloid accumulation and tau modifications at the diseased residues of the neurons (Janczura et al., 2018). This further demonstrates the potential value of HDAC drug therapy for patients beyond lower organism model results.

Similar significance of epigenetic acetylation patterns has also been observed in post-mortem human brain tissues, furthering the promise of HAT/HDAC related drugs in AD therapeutics. Although HDAC inhibition accounts for the greater representation of epigenetic therapeutics (meaning lower acetyl levels tend to be associated with AD neuropathology), some studies have identified an opposite pattern of epigenetic modification. Narayan et al. (2015) for instance, utilized immunolabeling and microarray analyses to demonstrate increased H3 and H4 acetyl levels in post-mortem AD inferior temporal gyrus and middle temporal gyrus brain tissues compared to normal brain tissue, along with compromised protein degradation mechanisms. Observed differences significantly correlated with tau, β-amyloid, and ubiquitin pathology, as they were only present in areas associated with pathology and were not identified in the control cerebellum tissues (Narayan et al., 2015). It should be noted, though, that post-mortem experiments tend to have smaller sample sizes than mouse model or cellular based analyses. Despite the contradiction with mouse models that emphasize deficiencies in acetylation rather than hyperacetylation, these results still demonstrate that there clearly exist dysregulations in epigenetic mechanisms in AD pathologies.

## DNA Methylation’s Potential in AD Therapeutics

DNA methylation is widely regarded as the most extensively studied epigenetic modification (Anderson et al., 2012). Although the focus was previously with regard to cancer (Laird and Jaenisch, 1996; Baylin and Herman, 2000), methylation has now taken a position at the forefront of Alzheimer’s disease research, and may provide insight into new therapeutic approaches to ease the neuropathology of this crippling disease.

Various studies now examine methylation patterns of numerous disease-associated genes to determine which genes have differential patterns upon pathology, either in the form of hypomethylation or hypermethylation. The same genes are also frequently studied in more than one bodily location, for instance hippocampal cells versus blood cells. *HOX* genes are of particular interest due to the critical role they play in neural development as they encode transcription factors responsible for neural patterning (Philippidou and Dasen, 2013). Recently, Smith et al. (2018) became the first study to demonstrate how extensively *HOX* gene differential methylation can span in AD patients. Their study has exhibited AD-associated hypermethylation across an extensive region (48 kb) of the *HOXA* cluster using epigenome-wide association in prefrontal cortex and superior temporal gyrus samples across three independent cohorts (Smith et al., 2018). In analyzing methylation dynamics in relation to pathology, one cannot simply apply a one-size-fits-all methodology, but rather must consider different genes having different patterns, with hypermethylation silencing of beneficial protective genes occurring at the same time that hypomethylation activation of problematic predisposition genes may be occurring. While hypermethylation of important developmental genes is observed in this region, so too is hypomethylation of *APP*, which results in greater levels of amyloid plaques and neuropathology (Gasparoni et al., 2018). DNA methylation dysregulation has also previously been observed in this region in Down syndrome individuals, which is of interest as many Down syndrome patients develop AD due to a duplicate of *APP* in the trisomy on chromosome 21 (Bacalini et al., 2015). Using post-mortem brain samples compared to standard aging profiles, Braak stage-associated methylation variations in both neurons and glia has further been identified in numerous other genes associated with AD progression, such as *MCF2L*, *ANK1*, *MAP2*, *LRRC8B*, *STK32C*, and *S100B* (Gasparoni et al., 2018).

Various aspects of neuropathology are now known to have links to differential methylation patterns. Immunoreactivity analyses of entorhinal cortex layer II, known for substantial AD pathology shows epigenetic dysfunction, particularly significant decrements (such as in 5-Methylcytosine and 5-methylcytidine) in neuronal immunoreactivity of all 10 of the epigenetic markers and factors studied by Mastroeni et al. (2010), including *PHF1/PS396*, *DNMT1* (major methyltransferase) and 6 components of *MeCP1/MBD2* methylation complex (*MTA2, HDAC1, HDAC2, p66α, RbAp48*, and *MBD2/3*). These results demonstrate an inverse relationship of DNA methylation markers and markers for late-stage tangles, as a PHF1 and PS396 are widely regarded as markers for neurofibrillary tangle formation (Mastroeni et al., 2010). In addition to the loss of methylation in neurons being associated with tangle formation, loss of methylation is also linked to increased expression of cell cycle genes (Jackson-Grusby et al., 2001; Mattson, 2003) and thus observed decrements in methylation in AD neurons could be linked to the aberrant re-entry into cell cycle and apoptosis observed in AD. One study even identified 11,822 hypermethylated CpGs in AD profiles (as well as 6,073 hypomethylated CpGs), with most of the hypermethylated sites being genes associated with cell-cycle associated processes (such as regulation of mitosis and phase transitions etc) as well as wnt-signaling involved in synaptic modulation and cognitive impairment, whereas hypomethylated sites were identified as genes involved in transcription factor binding, cofactor binding, and promoter binding (Gao et al., 2018).

As previously mentioned, location is also noteworthy when studying AD-related genes. When analyzing to see if genes associated with early onset AD are differently methylated, pyrosequencing of AD blood and brain samples have shown that only *RIN3* in blood cells exhibits significant hypomethylation for 7 CpGs (Boden et al., 2017). *RIN3* encodes a potassium-dependent sodium/calcium exchanger and is associated with cell signaling and neural development through synapse function and endocytosis roles by negatively impacting amyloid trafficking (Giri et al., 2016). In the same study that identified *RIN3* hypomethylation, no group-wide significant differences were observed for late-onset genes *PTK2β*, *ABCA7*, *SIRT1*, or *MEF2C* (although 1 CpG of *MEF2C* did have reduced methylation in one AD individual) (Boden et al., 2017). This suggests that early versus late-onset AD pathologies may not permit a universal epigenetic therapeutics solution. *TNF-α*, on the other hand, only shows significant hypomethylation in the cortex samples of AD patients but not in blood samples, showing that some of the epigenetic mechanisms being uncovered in AD pathology are only relevant to brain cells, not blood cells (Kaut et al., 2014), while others are only observed in blood cells (Boden et al., 2017). This hypomethylation at the promoter region of tumor necrosis factor has been linked to a suppression in its activity due to a lack of transcription factor binding (Pieper et al., 2008), which then leads to significant deficits in cognitive and synaptic function, as it triggers an accumulation of amyloid plaques (Buchhave et al., 2010).

Beyond the cognitive impairment and memory deficits that most people associate with AD, circadian rhythm disruptions are also highly prevalent with the majority of AD patients experiencing modified sleep/wake cycles, thermoregulation issues, and increased evening confusion (Satlin et al., 1995; Wu and Swaab, 2007). Upon examination of methylation, transcription, and expression of *BMAL1*, which is a known as a core component of the circadian rhythm clock and acts as a transcription factor that regulates the firing rate of hypothalamic suprachiasmatic nucleus neurons (Rudic et al., 2004), aberrant rhythmic methylation patterns significantly altering the expression of *BMAL1* have been observed in fibroblasts and post-mortem AD brain samples (Cronin et al., 2017). The promise of epigenetic therapies thus extends to circadian cycles and thermoregulation.

In addition to the promising potential of methylation-related epigenetic therapies for AD neuropathology, these therapeutic advancements can also aid beyond AD to help ease the suffering of other neurodegenerative disorder patients. Significant similarities are observed in differential methylation studies when AD samples are compared to other disorders including Bipolar Disorder (BD), Huntington’s, Parkinson’s, Vascular Dementia, and Lewy-bodies Dementia (Rao et al., 2012; Smith et al., 2019). Upon testing the CpG methylation of AD and bipolar disorder associated genes, as well as global DNA methylation and histone modifications in post-mortem frontal cortex of 20 patients with these neurodegenerative disorders (10 of each) AD and BD brains, many epigenetic similarities were observed. Global DNA hypermethylation and histone H3 phosphorylation is present in both illnesses, as well as hypomethylation at the *COX-2* promoter, hypermethylation at the *BDNF* promoter. CpG methylation of synaptic markers is present in both illnesses, but there is an increase in methylation of the synaptophysin promoter in AD only, while drebin hypermethylation is only present in BD. In addition to methylation variations, BD and AD present with an increase in mRNA and protein of neuroinflammatory markers (IL-1β, TNF-α, astrocytic, and microglial activation markers) (Rao et al., 2012). Such epigenetic similarities and the potential of multi-illness therapeutics are promising ventures to study as both disorders are similarly characterized with increased neuroinflammatory markers GFAP, CD11b, IL-1β, increased AA cascade cPLA2IVA, sPLA2IIA and COX2, and the loss of neurotrophic BDNF and pre-/post-synaptic synaptophysin and drebin (Rao et al., 2011). Beyond BD, bisulfite pyrosequencing demonstrates that *ANK1* hypermethylation is not only observed in AD, but is also observed in Huntington’s disease and Parkinson’s disease, whereas samples with Vascular Dementia or Lewy bodies Dementia also demonstrated *ANK1* hypermethylation, but only when they had coexisting AD-pathology (Smith et al., 2019). This further demonstrates that methylation-related epigenetic therapeutics could extend beyond just ameliorating AD pathologies.

To further explore the epigenetic methylation profile differences being observed in AD samples, some studies have even utilized twins to better characterize genetic risks. Reduced Representation Bisulfite Sequencing of monozygotic and dizygotic twin pairs to examine whether epigenetic profile differences associated with AD could be detected in the blood of participants sharing similar genetic risk profiles shows twin pairs contain epigenomic differences in AD pathology associated genes such as *ADARB2*, including differentially methylated sites in hippocampal cells rather than just blood cells (Konki et al., 2018). *ADARB2* mutant models are known to demonstrate memory and learning deficits, as well as synaptic impairments (Mladenova et al., 2018). Quantitative immunohistochemistry to study the levels of DNA methylation and hydroxymethylation in post-mortem AD patients’ brains has also shown significant decreases of 5-methylcytosine and 5-hydroxymethylcytosine in AD patients with similar results observed in an AD twin compared with the healthy twin. Furthermore, levels of methylation and hypermethylation had a negative correlation with hippocampal amyloid plaque levels and neurofibrillary tangles, meaning that reduced methylation in those same AD patients correlated with increased amyloid proteins and tangles, although it is unknown whether it was a causal or consequential event. It should be noted, though, that sample sizes were low with only 10 post-mortem AD samples, 10 post-mortem control samples, and only one set of twins (Chouliaras et al., 2013). Beyond the genetic risk profile, it is also important to better understand the environmental lifestyle risk profiles as well to ensure the most comprehensive knowledge of AD in order to most effectively target it (Eid et al., 2018). The largest study of DNA methylation-based aging (biological epigenetic profile age versus actual chronological age) to date utilized over 5000 individuals to assess genetic and environmental Alzheimer’s disease risk factors, which allowed for the identification of significant associations with regard to lifestyle risk factors rather than genetic factors. Body mass index, cholesterol levels, socioeconomic status, high blood pressure, and smoking behavior all were significantly associated with AD and age acceleration epigenetic profiles (McCartney et al., 2018).

Although differential methylation has been elucidated with various AD-associated genes, some studies have instead disputed the idea of DNA methylation involvement in AD progression in other AD-associated promoters. Nagata et al. (2018), for instance, provided evidence that there is no differential methylation observed at the NEP promoter of post-mortem AD brain samples (Nagata et al., 2018). NEP is a metalloprotease involved in the degradation of β-amyloid proteins, and known to be deficient in AD-pathologies where amyloid plaques accumulate (Turner et al., 2004). The precise downregulation mechanism of NEP in AD progression still remains to be explained. Overall, the evidence for methylation playing a role in AD progression and pathology exceeds any disputes, and thus presents a very strong case for exploring epigenetic therapeutics in the targeting of AD.

## Chromatin Remodelers and Other Histone Modifications in AD

### Chromatin Remodelers

While chromatin remodelers play a crucial role in regulating chromatin, there is currently a lack of information regarding the position that these enzyme complexes play in AD compared to that of other epigenetic mechanisms. That being stated, there does exist some data that shows the connection between chromatin remodelers and AD. For instance, current research has revealed that CHD5, a chromatin remodeler belonging to the CHD family, plays a critical role in Alzheimer’s. While most remodeling ATPases are expressed throughout the human body, CHD5 expression is confined to the brain (Potts et al., 2011). Moreover, the depletion of CHD5 impacts SWI/SNF, another family of chromatin remodelers. When depleted, CHD5 particularly impacts the subunits of SWI/SNF that are found in the brain by changing their expression levels. CHD5 has also been specifically linked to the genes implicated in Alzheimer’s, as CHD5 has been shown to directly regulate them (Potts et al., 2011). Thus, a strong connection of the role CHD5 and AD has been documented.

Additional studies have shown the potential relationship of other chromatin remodelers with AD as well. Microarray analysis has shown that “SWI/SNF related, matrix associated, actin-dependent regulator of chromatin subfamily a, may also be found to be associated with Alzheimer’s” (Guttula et al., 2012). Additionally, INO80, Proteasome, and RNAPII machinery have also been shown to be associated with Alzheimer’s disease, potentially via RNAPII degradation by INO80 (Poli et al., 2017). Further research will be needed to further confirm these connections.

### Other Histone Modifications: Phosphorylation

Other histone modifications have been seen to play a role in AD as well. Phosphorylation is a type of histone modification that can occur when a phosphate group is added on to the histone tails of the nucleosome. Research has shown that the H2AX protein in the nucleosome of astrocytes, a type of supportive nerve cell, is phosphorylated in response to double strand breakages in the DNA. When this occurs, there is a conversion of the H2AX protein into γH2AX. This conversion is specifically found in greater amounts in astrocytes located in the hippocampal and cerebral cortex sections of the brain. Interestingly, these regions are the same areas known to be impacted in AD (Myung et al., 2008). The above-mentioned studies were performed on brain tissue acquired from autopsied patients with AD. They indicate that the phosphorylation found in astrocyte DNA signifies chromosomal damage, which hinders its role in supporting surrounding neurons in the area.

Phosphorylation of another core histone protein has been observed as well. The core histone protein H4 has been shown to have significantly higher phosphorylation levels on Serine-47 in rats with levels of APP in their neuroblastoma cells compared to rats that were null in APP for these same types of cells. Experiments using tissue samples from the brains of AD patients confirmed these results, as high levels of phosphorylated H4 were observed. These findings are interesting as inhibition of H4 phosphorylation is now thought to be a potential means of protection against the pathological progression of AD as proposed by the authors (Chaput et al., 2016).

Yet another study suggesting the importance of phosphorylation in AD was published by Anderson and colleagues. Using transgenic mouse models that had increased amyloid deposits which mimicked the amyloid pathology that is characteristic of AD, they studied the phosphorylation of serine-57 and threonine-58 on H3, a histone greatly regulated by phosphorylation. Data showed a 40% reduction in serine-57 phosphorylation and a 45% reduction in threonine-58 phosphorylation in these transgenic mice compared to wild type. Additionally, there was a 30% reduction of the doubly phosphorylated serine-57 and threonine-58 sites. This decline in phosphorylation is likely thought to result in a more repressed chromatin structure, which in turn aligns with the epigenetic blockage exhibited in Alzheimer’s (Anderson et al., 2015). This again offers the possibility of using phosphorylation inhibition as a potential targeted therapy for AD. While more research is needed to determine if such therapies would even be a possibility, these studies highlight how the dysregulation of phosphorylation is yet another example of the important role that epigenetic mechanisms play in AD.

## Conclusion and Future Implications

In this review, we have concentrated on presenting an overview of the epigenetic dysregulation observed in AD. While the relationship between the role of impaired epigenetic modifications in Alzheimer’s has been a relatively recent one, the ever-increasing amount of research in this field has confirmed the importance of the connection. As this review has shown, the overall argument being built is a strong one, as epigenetic mechanisms, particularly those of DNA methylation and histone acetylation and deacetylation, show a clear dysregulation in AD when compared to the norm. It is critical to be able to target individuals that are most at risk for developing AD and thus try to proactively treat them as soon as possible. There are so many variables to be considered that this task can appear overwhelming. There is no one-and-only cause for this debilitating disease, but rather a series of interactions amongst circumstances, the environment, and genomes. Even within genomes there are multiple variables to consider, as age-associated genes (Desikan et al., 2017) and epigenetics (Lemche, 2018) can both influence potential buildup of neuropathological peptides and plaques. Epigenetic profiles can vary throughout an individual’s lifetime, especially since beyond age, factors such as stress, smoking, alcohol use, and diet can all affect epigenetic expressions and neuropathologies (Lövblad et al., 1997; Delgado-Morales et al., 2017). This may suggest that healthier life-choices, such as educated dieting and exercise routines, beyond prescription drug therapeutics may also be of importance in the treatment and prevention of progression in AD.

Based on the current research, in addition to lifestyle changes, we have specifically highlighted the potential for epigenetic therapies that can be used to help target the disease. This is of great significance, since as of now there are no known cures for AD that can treat the disease or even delay its process (Holtzman et al., 2011; Lindsley, 2012; Mitra et al., 2019). Although there is still some sporadic controversy around epigenetic studies with regard to the involvement of particular genes in AD, the overwhelming evidence in support of epigenetic connections warrants further attention. We believe that with more research the relationship will only become clearer and the development of more specific targeted therapies will arise to aid in the treatment of AD.

## Author Contributions

ME and GS contributed equally to the production of this work, writing and revising, as well as approving the submitted version.

## Conflict of Interest Statement

The authors declare that the research was conducted in the absence of any commercial or financial relationships that could be construed as a potential conflict of interest.
